# Acknowledgment to the Reviewers of *Current Issues in Molecular Biology* in 2022

**DOI:** 10.3390/cimb45020069

**Published:** 2023-01-29

**Authors:** CIMB Editorial Office

**Affiliations:** MDPI AG, St. Alban-Anlage 66, 4052 Basel, Switzerland

High-quality academic publishing is built on rigorous peer review. *CIMB* was able to uphold its high standards for published papers due to the outstanding efforts of our reviewers. Thanks to the efforts of our reviewers in 2022, the median time to first decision was 17 days and the median time to publication was 39 days. Regardless of whether the articles they examined were ultimately published, the editors would like to express their appreciation and thank the following reviewers for the time and dedication that they have shown *CIMB*:
Abate, Maria LorenaMaggi, FedericaAbbate, Jessica MariaMahmod, Asma IsmailAbdullah, AbdullahMahmoudi, NiloufarAbend, MichaelMahpara, ShahzadiAcar, NurayMaier, Stelian SergiuAceto, Gitana MariaMaj, Mary C.Adamakis, Ioannis-DimosthenisMalatesti, NelaAdams, Brian D.Malhotra, KaranAdams, Sandra D.Malik, AnuragAgudelo-Suárez, Andrés AlonsoMallamaci, RosannaAguiar, CristianeManai, FedericoAhmad, AshfaqMarasco, DanielaAhmar, SunnyMarchetti, AlessandraAhn, Ji HyeonMarco, Eva M.Akimov, Mikhail G.Marković, ZoranAkolkar, HimanshuMarowsky, AnneAktepe, Turgut E.Marsano, René MassimilianoAl-alem, UmaimaMarsili, StefaniaAlalwan, Tariq AbdulKarimMartín Casanueva, Miguel AngelAl-Amri, Issa SulaimanMartínez Beamonte, RobertoAlavi, MehranMartínez-Gómez, PedroAl-Bari, Abdul AlimMartínez-Hernández, JoséAlbert, Daniel ArthurMartinez-Pastor, FelipeAlcaro, StefanoMartino, Piera AnnaAlgamal, ZakariyaMartu, IoanaAlhasnawi, ArshadMatei, IrinaAli Raza, MuhammadMatin, MahbubulAli, AhmedMatsubara, EriAlizargar, JavadMatsubara, KazumiAllegaert, KarelMatsukuma, SusumuAlloatti, GiuseppeMatthew, RätsepAlnuqaydan, AbdullahMatwijczuk, ArkadiuszAlter, SvenjaMauk, Michael G.Alves, FranciscoMaxim, AurelAlves, RosanaMazloum, AliAmaral-Silva, Gleyson Kleber DoMazumdar, PurabiAmarowicz, RyszardMcAlinden, Kielan DarcyAmerizadeh, AtefehMcBeath, ElenaAnamthathmakula, PrashanthMcEwan, William A.Andre Alberto, WeberMedrado, Alena Ribeiro Alves PeixotoAnshori, IsaMeerson, AriAnver, ShajahanMehana, ElsayedArab, Mohammad MehdiMehraeen, EsmaeilAraya-Maturana, RamiroMeliţ, Lorena ElenaArcher, TrevorMeng, XiaoxiAriadna Thalía, Bernal-MercadoMenghini, LuigiArun, YuvarajMenon, Archita VenugopalAshihara, EishiMesquita, CristinaAshraf, Muhammad AleemMesserer, David Alexander ChristianAshraf, UmairMiertus, StanislavAssiri, AbdullahMilatz, SusanneAstakhova, KiraMiller, Catherine M.Asunción Nadal, Víctor De LaMimura, JunseiAtrash, ShebliMina, AbhaAttaie, RahmatMinea, RaduAttwa, Mohamed W.Minkina, TatianaAureliano, ManuelMiotto, BenoitAvram, MarioaraMiricescu, DanielaAyaz, AhmetMironeasa, SilviaAzam, FaizulMiroshnikova, Valentina V.Azinheira, Helena G.Mishra, Jay S.Babulak, EduardMishra, MeerambikaBach, Thomas J.Mishra, VibhorBadescu, Minerva CodrutaMisra, AnuragBakht, MartinMisztak, PaulinaBakillah, AhmedMitarai, SatoshiBalajthy, ZoltánMitsuyama, SusumuBalestrieri, EmanuelaMiyamoto, KojiBalnytė, RenataMiyasita, KazuoBaltina, TatyanaMizutani, TaketoshiBanerjee, KalpitaMnich, KatarzynaBao, RuiyangModos, DezsöBao, YupingMohsin, MuhammadBär, SéverineMoise, Adela RamonaBaranova, EkaterinaMola, Maria GraziaBar-Haim, ErezMolina-Cerrillo, JavierBarthet, MichelleMoloudizargari, MiladBassanini, IvanMondego, Jorge Maurício CostaBastiaanse, HeloiseMoneim, DiaaBatori, RobertMonget, PhilippeBattistelli, MichelaMorais, MychelBeberok, ArturMorak-Młodawska, BeataBednarska-Czerwińska, AnnaMorato, Manuela Sofia RodriguesBehzadi, PayamMori, MasaakiBeier, Marcel PascalMori, ShogoBelitsky, Jason M.Mori, TetsushiBeltrami, RiccardoMorita, KyojiBermúdez Echeverry, MarcelaMornar, AnaBeyzaei, ZahraMoschovi, MariaBhukel, AnuradhaMotamed, CyrusBhyan, SalmaMotiani, RajenderBianchi, GiacomoMouzaki, AthanasiaBianco, Maria RitaMozdarani, HosseinBielenica, AnnaMrsa, VladimirBieniek, AnnaMuleo, RosarioBilir, NebiMüller, GünterBilteanu, LiviuMuntean, SorinBiscaglia, GiuseppeMurata, KazumotoBiswas, RoopaMurata, SoichiroBjørklund, GeirMurzakhanov, Fadis F.Blanco, AntonioMuthusamy, VigneshBlanton, CynthiaMykhailenko, OlhaBody-Malapel, MathildeMylona, PhotiniBoldura, Oana-MariaNabavi, Seyed MassoodBondžić, Aleksandra M.Nair, JayakumarBoonen, MarielleNair, Prakash M GopalakrishnanBora, AlinaNakayama, YoshitakaBørsting, ClausNakazawa, MitsuruBottari, SergeNalepa, IrenaBoufidou, FotiniNallasamy, PalanisamyBouissane, LatifaNaoe, TomokiBoussios, StergiosNara, AtsukiBoyarskiy, Vadim P.Narayana, Vinod K.Bozic, JoskoNaryzhnaya, NataliaBrattsand, MariaNatali, Maria Raquel MarçalBreitenfeld Granadeiro, LuizaNatalicchio, AnnalisaBroecker, FelixNaumov, Anton V.Broggini, ThomasNawade, BhagwatBrullo, ChiaraNde, Pius N.Brusgaard, KlausNées, MatthiasBryant, William BartNenu, IulianaBucur, AlexandruNeri, TommasoBugarin, AlejandroNeves, Cristina BettencourtBulatov, EmilNguyen, TrangBullon, PedroNi, WeiBüttner-Teleagă, AntjeNicosia, AldoCabrera Orefice, AlfredoNielsen, Vance GirardCadamuro, MassimilianoNikolaev, ViacheslavCalucho, MaiteNishida, HarutoCampagna, RobertoNitulescu, MihaiCampbell, Lia H.Nixon, Daniel W.Cao, DongdongNobrega, FranklinCao, HongnanNogara, PabloCariati, IdaNoguera-Velasco, José A.Carmen, MejíaNoor, RashedCarrizzo, AlbinoNoreen, SobiaCarter, WayneNovikov, FedorCaruana, IgnazioNovopashina, DaryaCarvalho, MárciaNowicka, GrażynaCaspari, ThomasObara-Michlewska, MartaCastro, HeribertoOcchipinti, SergioCastro-Sánchez, LuisÖçsoy, İsmailCastrucci, Ana MariaOda, MasatakaCazacu, IrinaOettmeier, ChristinaCervellati, FrancoOgasawara, ToruChan, AndrewOgawa, ShinichiroChan, Ming HsienOğuzoğlu, Tuba ÇiğdemChang, Che-ChienOkazaki, YasumasaChang, Ching-JinOkpala, Charles Odilichukwu R.Chang, Kyung-SooOlaru, Octavian TudorelCharles Richard, John LalithOliviu, VostinaruCharles, Albert LintonOmar, Syed HarisChathuranga, KiramageOpoka-Winiarska, ViolettaChatzis, LoukasOroz, JavierChen, Edward S.Ortiz, Ana GodoyChen, RunpuOrton, Thomas J.Chen, Sung-LangOuni, MeriemChen, WeiOzawa, KoyaChen, XiÖzkan, SelinChen, Yi-ChengPacini, MatteoChen, Yi-JuPacławski, AdamChen, YTPaczos-Grzeda, EdytaChen, Yuh-shuenPagano, Allan F.Cheng, Juei-tangPal, DebjaniCheng, JunruiPaliwal, RajneeshCheong, Yuen-KiPalma, Tina DiChernonosova, VeraPandey, ManojChetty, Dharshnee RamaPandey, NootanChevalier, FrançoisPandey, SudhirChiarelli, RobertoPandey, Swaroop KumarChinnam, Naga BabuPantano, LiciaChintapalli, Sree V.Papaccio, FedericaChittepu, Veera C.S.R.Papaccio, GianpaoloChowdhury, DebabrataPapachristodoulou, AlexandrosChrcanovic, BrunoPapadakis, Andreas IoannisChu, XiakunPapadopoulos, SerafeimChyau, Charng-CherngPapaioannou, MariaCiampaglia, RobertoParadowska-Stolarz, AnnaCiećwież, SylwesterParanthaman, Balasubramani SubramaniČipak Gašparović, AnaPark, Jung-hyunCoertse, JessicaParsons, PeterCohen, GeraldPasi, FrancescaCollart-Dutilleul, Pierre-YvesPastore, NunziaCombarnous, YvesPatel, NibeditaComitato, RaffaellaPattanayak, Subrat KumarCopolovic, Dana MariaPaturi, Durga KalyaniCosacak, Mehmet IlyasPaul, SourenCostache, Raluca SimonaPedraza Chaverri, JoséCoțovanu, IonicaPelivan, IvicaCotterill, SuePellegrini, Massimiliano MatteoCovantev, SergheiPelosi, EmanueleCrocè, Lory SaveriaPeng, HaixinCrosio, ClaudiaPeraldi, PascalCruz, AndreaPerdomo, JuanCruz-Sosa, FranciscoPérez Torres, ArmandoCuca, Luis EnriquePérot, PhilippeCucinotta, MaraPerrotta, AdolfoCui, HongzhuPerše, MartinaCui, Yan-HongPervaiz, TariqCurrò, MonicaPeters, LindaCusick, Matthew F.Pettas, EfstathiosCzapla, JustynaPhelix, Clyde F.Czerwik-Marcinkowska, JoannaPhilippe, AurélieD’Antona, NicolaPiacentini, LuciaDa Hora, Gabriel Costa A.Piccin, AndreaDabelic, SanjaPicimbon, Jean-FrançoisDar, Wasim A.Pinho, Brígida RibeiroDaras, GerasimosPini, Luigi AlbertoDavoodi, AliPinto, Sandra N.Dayem, Ahmed AbdalPisani, LauraDe Brevern, Alexandre G.Pisapia, PasqualeDe Sanctis, Juan BautistaPistamaltzian, NikolaosDegl’innocenti, DonatellaPittenger, Mark F.Del Casale, AntonioPivetta, ElianaDe-la-Torre, PedroPizzolanti, GiuseppeDeligiannidou, Georgia-EiriniPłonka, PrzemysławDhar Dwivedi, Shailendra KumarPłoszaj, TomaszDi Bartolomeo, SabrinaPlotnikov, Egor Y.Di Donato, MarziaPoirot, MarcDi Gennaro, FrancescoPolakowski, Nicholas J.Di Paola, RosannaPolis, BaruhDiercks, Björn-PhilippPonti, DonatellaDixit, ShivaniPopovici, VioletaDobhal, ShefaliPospelov, AntonDoina Vrabie, CameliaPotaczek, Daniel P.Doke, MayurPotęga, AgnieszkaDomingo, GuidoPotocny, Andrea M.Domingues, CátiaPoyraz, SametDomingues, Douglas SilvaPratesi, FedericoDong, JiePriolo, ManuelaDongtak, JeongPrisinzano, ThomasDonnini, SandraPrzemysław, NucDovnik, AndraẑPrzystupski, DawidDrozd, ArletaPuppo, MargheritaDryga, AnatolyPushparaj, SamuelDuarte-Escalante, EsperanzaPuzansky, Roman K.Duggan, Brendan M.Pyka-Pająk, AlinaÐulović, AzraQazi, Izhar HyderDunaeva, Marina V.Quan, Zhe-ShanDurgo, KsenijaQueiroz, GlóriaDutta, PranabanandaRacoviceanu (Băbuță), RoxanaDyakonov, VladimirRacz, IldikoDziak, RosemaryRadan, MilaEhrenfeld, PamelaRaghav, Pawan KumarEl Khalloufi, FatimaRagusa, Maria AntoniettaEleftheriadis, TheodorosRahat, Michal A.Elghobashi-Meinhardt, NadiaRaimondi, VittoriaElhenawy, Ahmed A.Rajan, AnubamaElkady, AbeerRakić, AleksandarElmann, AnatRakitin, Oleg A.Elsherbiny, NehalRamalho, Maria JoãoEndre Károly, KristófRamaswamy, BharathEnomoto, HirayukiRampias, TheodorosErdoğan, AyşegülRanzato, EliaErgin, VolkanRapacz, MarcinErrigo, AlessandraRaţă, Delia MihaelaEsmerino, Luis AntônioRay, SupriyoEspinel-Ingroff, Ana V.Raza, Sayed Haidar AbbasEsposito, MassimilianoReda, RodolfoEstêvão, M. DulceReynes, BarbaraEsworthy, Robert StevenRibarits, AlexandraEyben, Finn Edler VonRicciarelli, RobertaFabrizi, CinziaRinaldi, IkhwanFacchini, SergioRinaldi, Maria MadalenaFagone, PaoloRíos-Silva, MónicaFalconi, MaurizioRitter, AlexanderFaleiro, Maria LeonorRius-Pérez, SergioFalzone, LucaRizzo, AlessandroFang, QingmingRoberto, Sergio RuffoFarina, LorenzoRodin, Andrei S.Farré, NúriaRodríguez-Landa, Juan FranciscoFeliciello, IsidoroRodríguez-Reséndiz, JuvenalFendrihan, SergiuRoeth, DanielFeo, SalvatoreRogers, Robert S.Ferini, GianlucaRoghanian, MohammadFernández, Maria VictoriaRolinec, MichalFernández-Coll, LlorençRomano, CorradoFernández-Gil, Beatriz I.Romeo, Maria AneleFerrante, PatriziaRomero, Carlos HFerraz, Renato BarbosaRomero, Marta R.Ferreira De Oliveira, José Miguel P.Rondón, CarmenFerreira, FernandoRose, ShannonFerreira-Santos, PedroRoselli, MarioFerrer, Rubén A.Rosero-Garcia, DorisFersing, CyrilRossi, Franco RubénFibach, EitanRossignoli, FilippoFinelli, CarmineRougé, PierreFinkina, EkaterinaRueda, EdgarFionda, BrunoRuggiero, EmanuelaFoffa, IleniaRump, KatharinaFogacci, FedericaRusu, AuraFontana, GianfrancoRychel, SandraFornalski, KrzysztofRyu, Kyoung-SeokFortofoiu, MariaRzepka, ZuzannaFousteris, ManolisSabater, LidiaFrasson, IlariaSadanandan, JayanarayananFrietze, SethSadhukhan, TamalFritzler, Marvin J.Saeed, MohdFthenakis, GeorgeSaeki, KumikoFujihara, HisakoSaha, ShekharFujitani, YoshioSahgal, PranshuFukumoto, YoshihiroSahu, Pramod KumarFung, Alan Chi ChungSajjad, MuhammadFunk, RichardSak, Jarosław J.Fusco, RobertaSalem, KhaledG. Gaidin, SergeiSambri, AndreaG. Hegazi, AhmadSanchez, SusanGadila, ShivaSandri, GildaGagat, PrzemyslawSannigrahi, AchintaGahlaut, VijaySansone, AnnaGaikwad, ShreyasSantiago, LeandroGallego, MonicaSantilli, FrancescaGamarra, LionelSantoro, GiorgioGangadaran, PrakashSantos-Jimenez, ZurisadayGao, ShiqiangSanz-Garcia, CarlosGarcía-Mesa, YolandaSarang, ZsoltGarcía-Otín, Ángel LuisSarmay, GabriellaGates, ColinSarshar, MeysamGautam, UmaSawai, HirofumiGauvreau, GailSawicki, JakubGawłowska, MagdalenaScala, AlessandroGazaryan, Irina G.Schapher, MircoGazdhar, AmiqSchlicker, EberhardGhartey-Kwansah, GeorgeSchling, PetraGhelardoni, SandraSchmidt, MarcelGhiciuc, Cristina MihaelaSchmitz-Linneweber, ChristianGhimire, Bimal KumarSchneider-Stock, RegineGhorbani, AbozarSchusser, Gerald FritzGhosh, ArunavaScudiero, OlgaGhosh, SoumenSegat, LudovicaGiacometti, CinziaSeger, RonyGiannakas, ArisSegovia, CristinaGieczewska, KatarzynaSeifi, SorenGil, Andrea Paola RojasSekimizu, KazuhisaGlazińska, PaulinaSelvaraj, ChandraboseGluhovschi, CristinaSen, AnandoGnanasekaran, PrabuSenthil Kumar, RajuGnyba, MarcinSepúlveda, JuanGokuldass, AishwaryaSerapinas, DanieliusGomes, Maria SaloméSergeeva, YanaGomez, RameshSerrano, Dolores R.Gonçalves, JorgeSethunath, VidyalakshmiGong, ShiqiangSeto, ShigekiGonzalez Prendes, RaynerSewduth, RajGonzález-Minero, Francisco JoséSganzerla, WilliamGoormaghtigh, FrédéricShahrajabian, Mohamad HesamGordon, Darren M.Shaik, Haq AbdulGoris, TobiasShaito, AbdullahGote, VrindaShalata, WalidGoverna, PaoloShan, WeiranGrabarek, Beniamin OskarShao, PengGraber, Zachary T.Sharara, FadyGrabowska, MartaSharma, GauravGredic, MarijaSharma, Ravi KumarGregorini, MarilenaShepelev, MikhailGrigore, MihaescuShibukawa, YoshiyukiGrody, Wayne W.Shiina, MarisaGrześk, GrzegorzSiciliano, GabrieleGu, WeiSicko, Robert J.Gueniche, AudreySieber, FritzGültas, MehmetSikora, MariuszGumieniczek, AnnaSilva-Espinoza, Brenda A.Gummidi, LalithaSimon, JorgeGUO, Zong ShengSimon, LizGusar, VladislavaSimpson, AnnGuth, AmandaSingh, Chandra MohanGutierrez-Alanis, DoloresSingh, DevendraGutierrez-Merino, CarlosSingh, HarbinderHa, Ki-TaeSingh, HarpalHadi, AmirSingh, KamalendraHadzic, StefanSingh, PradeepHameed, KhalidSingh, SimaHammad, LamiaaSingla, NidhiHammer, AstridSinha, AbhilashaHannan, AbdulSinha, RanjitaHao, FanfanSinha, Sudarson SekharHasan, NurhasniSioga, AntoniaHasegawa, ShinyaSiristatidis, CharalamposHasegawa, YasushiSita, GiuliaHashimoto, MakotoSkorokhod, OleksiiHermann, EmmanuelSlim, SmaouiHerrera-Luis, EstherSmereka, JacekHervey, William JudsonSmidt, Eric De CamargoHeshmati, JavadSmith, StevenHeyse, AlexSoengas, Raquel G.Ho, Thanh-TamSolano, FranciscoHoang, Huy-DungSol-Foulon, NathalieHolmager, PernilleSolovyev, AndreyHoogduijn, Martin J.Somanath, Payaningal R.Horai, YoshiroSomlyai, GáborHosokawa, YoshitakaSoRelle, Jeffrey A.Hosomi, RyotaSorg, OlivierHouyel, LucileSorrentino, RosalindaHsu, Bang-geeSosa, SilvioHu, MengleiSoto-Hernández, MarcosHuang, Cheng-YangSousa, Susana R.Huang, QiangSouza Júnior, Manoel TeixeiraHublitz, PhilipSpekker, EleonóraHuey-Shan, HungSpissu, YleniaHuh, Sung UnSrivastava, RakeshHukowska-Szematowicz, BeataSrivastava, SewetaHume, Michael E.Stachler, Matthew D.Humińska-Lisowska, KingaStaudacher, ErikaHung, Yao-ChingSteele, ColinHuppi, KonradStefanucci, AzzurraHussein, Hebat-Allah A.Stimpfel, MartinIbrahim, IslamStobiecka, MagdalenaIbrahim, SabrinStoccoro, AndreaIelciu, IrinaSubramaniyan, BoopathiIkegami, HidetoshiSuda, GokiIldikó, TarSun, Yu H.Im, Dong SoonSundararajan, VigneshIm, SunSuvarna, KruthiIonescu, Răzvan AdrianSuzuki, AkikoIrannejad, HamidSuzuki, HideoIsath, Ameesh M.Sveshnikova, AnastasiaIshtiaq, MuhammadSymanowicz, BarbaraIslam, Md. AmirulSymonds, Dr. VaughanIsreb, AbdullahSzczerba-Turek, AnnaIto, ShosukeSztacho, MartinIwai, AtsushiSzymanska, MagdalenaIyer, SoumyaSzymczak-Pajor, IzabelaIzdebska, MagdalenaTabęcka-Łonczyńska, AnnaIzumi, KenjiTada, TakuyaJabłońska-Trypuć, AgataTahir, AyeshaJaehne, Anja KathrinTakagi, KiyoshiJain, NeerajTakamatsu, ShinjiJameel, M.Tan, Choo HockJankovska-Bortkevič, ElžbietaTanaka, TakujiJatuworapruk, KanonTanaka, YoshimasaJelińska, AnnaTang, Bor LuenJembrek, Maja JazvinšćakTang, YuJerzak, Malgorzata MariaTanislav, ChristianJesudhasan, PalmyTao, LitaoJin, ZhuqiuTayebati, Seyed KhosrowJorge-Mora, AlbertoTéllez-Valencia, AlfredoJovanovic, MirnaTemel, AslihanJózefczyk, AleksandraTenenbaum, LilianeJurenas, DukasTenopoulou, MargaritaJurowski, KamilTerán, CarmenKabakov, Alexander E.Teti, GabriellaKabekkodu, Shama P.Tewari, DeveshKadiam, Chinna Venkata SubbaiahThai, Khac-MinhKadziński, LeszekThakur, MeenakshiKai, KeitaTheotokis, PaschalisKalas, WojciechThiruvengadam, MuthuKalia, RaghavThompson, AndrewKamisah, YusofThummer, Rajkumar P.Kanczler, Janos M.Thuru, XavierKang, EunjuTiwari, RahulKang, Jae SeungTiziani, StefanoKang, Sang GuTogashi, YuichiKaradag, BülentToledano-Magaña, YanisKaren, KeeshanTomaszewska, EwaKaroubi, GolnazTomiczak, KarolinaKarwowski, BoleslawTony, HaighKashiwagi, AkikoTopin, JeremieKaur, GaganpreetTorices, SilviaKaushik, AbhinavTorres-Lagares, DanielKavsak, PeterTorti, MarinKawai, GotaTouraud, DidierKawai, TatsuoTran-Nguyen, Viet-KhoaKawakami, HiroshiTrumbić, ŽeljkaKe, Chien-ChihTsai, I-JungKhallouki, FaridTsai, RongkungKhan, BibiTsaousidou, EvaKhan, Muhammad Iqbal R.Tsim, KarlKhan, Naqib UllahTsitsopoulos, Parmenion P.Khan, ShahanshahTucci, Felicia AnnaKhan, ShahidTuvikene, RandoKhanh Le, Nguyen QuocTwelker, J. DanielKhapchaev, AskerTyurin, Anton P.Khotib, JunaidiTzounakas, Vassilis L.Khurram, MuhammadUddin, Md JamalKiiker, RiinuUkoha, Winifred ChinyereKim, DongGwanUmemura, NaokiKim, Ho BangValente, Neusa Yuriko SakaiKim, HyojungValentini, VirginiaKIM, HYUNGJINValentovic, MonicaKim, Kyung-MinValori, ChiaraKim, NayoungValtuille, RodolfoKim, Sung EunVanella, LucaKim, Sung-HoonVaradarajan, SudhaharKim, Sung-Kun (Sean)Varshney, AkhilKitzmüller, ClaudiaVelazquez-Salinas, LauroKmiec, Eric B.Velázquez-Sánchez, ClaraKobayashi, NoritoshiVelikova, TsvetelinaKobiela, TomaszVercellin, Alice VerticchioKobierzycki, ChristopherVerma, Praveen C.Kodama, HiroakiVicaș, Laura GrațielaKoeffel, ReneVidican, RoxanaKõks, SulevViennet, ThibaultKöksal, EdaVietri, Maria TeresaKolb, PeterVillalpando-Aguilar, Jose LuisKontos, FilipposVillanueva-Paz, MarinaKorbecki, JanVirgili, FabioKorga, AgnieszkaVishnyakov, Innokentii E.Korres, George S.Vivas-Reyes, RicardoKosan, ChristianVodnar, Dan C.Kosmalski, MarcinVolante, AndreaKostara, Christina E.Volpe, AndreaKotlowski, RomanVorobyeva, MariyaKotov, Alexey A.Vorster, MarizaKotula-Balak, MałgorzataWagner, KayKoustas, EvangelosWagner, Kay DietrichKouttab, NickWaleron, KrzysztofKrackhardt, AngelaWalory, JarosławKraft, RobertWan, LeiKrawczynski, KamilWang, Chi-YoungKrawczyński, Maciej RobertWang, Chrong-ReenKrishnasamy, GopinathWang, HaoKrok-Borkowicz, MalgorzataWang, HongyuKulbacka, JuulitaWang, HuafengKulkarni, ArpitaWang, JinrongKumar, AwanishWang, JixiaKumar, ManuWang, XianwenKumar, NareshWang, ZhijunKumar, SugandhWasilewska, EwaKumari, AnjuWawrocki, SebastianKundu, KiranWelsh, JaneKurmyshkina, Olga VadimovnaWelsh, MichaelKuzmich, AlexeyWen, JianjunKweon, Oh-KyeongWen-Chi, YangLaboisse, Christian L.Wilhelm, KerstinŁagowski, DominikWinkler, JanLaing, William A.Wiraja, ChristianLajayer, Behnam AsgariWong, Sok KuanLakota, JanWrześniok, DorotaLal, MohanWu, Cheng-LinLampridis, SavvasWu, Chung-ChunLankadasari, Manendra BabuWu, HongminŁapińska, BarbaraWu, Ping-HsunLaratta, BrunaWu, Tzong-YuanLattanzi, GianlucaWu, XinyanLavrov, AndreyWu, Yi HuiLázaro-Diéguez, FranciscoWyska, ElżbietaLazutka, JuozasXia, ZhiqiuLecewicz, MarekXu, JianqiangLedin, TorbjörnXu, WenyanLee, BrendaXu, XuejiaoLee, Dong-SeolYadav, AmarishLee, Hsin-ChenYadav, DhananjayLee, Jae-WonYamasaki, MasahiroLee, KwanghyunYang, Jiann-JouLee, Meng-JenYang, JingjingLee, Sung-hoYang, LimingLee, Sung-JaeYang, LiyanLei, SaifeiYang, MengquanLeisegang, Matthias S.Yang, Shun-FaLener, MatteoYang, Wei-hsiungLenzi, PaolaYang, Xiang-LeiLeonhardt, RalfYang, ZetianLeoni, ValerioYarwood, StephenLeontopoulos, StefanosYazawa, TakashiLešták, JánYen, Chia-HungLevante, AlessiaYesilkanal, Ali EkremLévêque, NicolasYílmaz, SerkanLewinska, AnnaYong, Miing-TiemLi, QianYoo, Yung ChoonLi, Sing-ChungYoshida, KumiLiao, Wei-BiaoYoshida, ManabuLin, HaishuYoshino, HironoriLin, RuweiYu, ZhigangLinial, MichalYuan, ShuiqiaoLionetti, VincenzoZandi, AshkanLiu, DongZaravinos, ApostolosLiu, Eric MinweiZavala-Tecuapetla, CeciliaLiu, Shing-HwaŻebrowska-Nawrocka, MartaLiu, WeisiZeleny, ReinhardLiu, ZhongweiZhang, DuoLlama-Palacios, AranchaZhang, ErshuaiLobo, María Val ToledoZhang, FenghuaLoboda, AgnieszkaZhang, JinweiLópez-Rosas, ItzelZhang, QuanweiLozanovski, VladimirZhang, RudianLozo, JelenaZhang, ShengLu, Hsueh-JuZhang, TiantianLu, Mei-ChinZhang, WenhengLu, Pei-LuenZhao, HuLu, XiyuanZhao, JingyiLucacchini, AntonioZhao, LinboLucaciu, RoxanaZheng, HaifengLucarelli, GiuseppeZhgun, AlexanderLulli, ValentinaZhong, WeiLupi, Saturnino MarcoZhou, HuifenLuzina, OlgaZielske, StevenMa, ChiZiemniak, MarcinMa, LiangZińczuk, JustynaMachnik, PeterZnamirowska, AgataMadonna, GabrieleZubareva, Ekaterina V.Magdy, MahmoudZwaan, C. Michel


